# Multiscale Numerical Modelling and Structural Design of Bulk Heterojunction Nanocomposites for Organic Photovoltaics: From Molecular Interfaces to Device Optimization

**DOI:** 10.3390/ma19153261

**Published:** 2026-08-01

**Authors:** Jie Dong, Ziyan Guo, Wei Hao, Hanying Li

**Affiliations:** MOE Key Laboratory of Macromolecular Synthesis and Functionalization, International Research Center for X Polymers, ZJU-YST Joint Research Center for Fundamental Science, Department of Polymer Science and Engineering, Zhejiang University, Hangzhou 310027, China; 12429034@zju.edu.cn (J.D.); 22529024@zju.edu.cn (Z.G.); hanying_li@zju.edu.cn (H.L.)

**Keywords:** organic photovoltaics, bulk heterojunction, nanocomposite, multiscale modelling, machine learning, donor–acceptor interface, morphology optimization, structural design

## Abstract

Bulk heterojunction (BHJ) active layers in organic photovoltaics (OPVs) are nanostructured composites in which electron-donating and electron-accepting semiconductors form interpenetrating phases for exciton dissociation and charge transport. The power conversion efficiency (PCE) of these organic-organic nanocomposites is governed by structural features spanning multiple length scales: molecular packing and energy-level alignment at donor/acceptor (D/A) interfaces, phase-separation morphology and crystallite connectivity, and thin-film optical and charge-transport characteristics. Rational design of high-performance OPV nanocomposites requires multiscale numerical modelling that bridges quantum chemistry, mesoscale morphology simulation, and device-scale optoelectronic modelling. This review surveys and critically compares recent advances in the structural design and numerical simulation of OPV BHJ nanocomposites. At the molecular scale, we examine density functional theory and non-adiabatic molecular dynamics approaches for resolving charge-separation driving forces, interfacial energy-level alignment, and exciton dynamics. At the mesoscale, we discuss molecular dynamics, kinetic Monte Carlo, and electronic coarse-graining methods for describing phase separation, crystallization kinetics, morphology evolution, and charge transport. At the device scale, we review exciton-diffusion, optical transfer-matrix, and drift-diffusion models that quantitatively link morphology to photovoltaic performance metrics. The review also evaluates how machine learning, high-throughput screening, surrogate models, and generative design accelerate donor–acceptor selection and morphology optimization, while distinguishing benchmark predictions from experimentally validated design rules. Across these scales, we compare the strengths, assumptions, and validation limits of the principal modelling approaches. Finally, we highlight emerging multiscale integration frameworks, including sequential parameter-passing pipelines and differentiable digital-twin concepts. By framing OPV BHJ layers as nanocomposites whose performance bottlenecks map onto composite-design challenges such as interface integrity, phase connectivity, multiscale charge transfer, and degradation-aware design, this review connects OPV modelling with broader structural-composites thinking for next-generation organic solar cells.

## 1. Introduction

### 1.1. Bulk Heterojunction Active Layers as Nanostructured Composites

Organic photovoltaics (OPVs) have undergone rapid performance advances in the past five years. Reported power conversion efficiencies (PCE) values for single-junction organic solar cells now exceed 20% in multiple independent reports [1,2,3]. This progress narrows the gap with established photovoltaic technologies while retaining the intrinsic advantages of organic semiconductors: solution processability, mechanical flexibility, semitransparency, and tunable optical absorption [4,5]. Most high-performance solution-processed OPVs use a bulk heterojunction (BHJ) active layer—a bicontinuous interpenetrating network of electron-donating (D) and electron-accepting (A) organic semiconductors, typically on the order of 100 nm thick, in which the nanoscale morphology strongly influences exciton generation, diffusion, dissociation at D/A interfaces, and charge transport to electrodes [6,7].

From a structural materials perspective, the BHJ active layer can be treated as a structurally tunable nanocomposite. It consists of two distinct organic phases—each with its own electronic structure, packing motif, crystallinity, and thermal behaviour—often mixed on the tens-of-nanometers scale to increase interfacial area while maintaining percolating pathways for charge extraction [4]. The structural design of this composite is demanding: the morphology must simultaneously support exciton diffusion over distances comparable to the exciton diffusion length, provide molecular-level D/A contact with appropriate energetic offsets, and maintain continuous pathways of each phase to the respective electrodes. These requirements map onto composite-design challenges such as interface integrity, phase connectivity, multiscale charge transfer, and structural stability during operation.

This composite-design perspective motivates the multiscale framework developed in the following sections, where molecular interfacial structure, mesoscale morphology, and device-scale optoelectronic response are treated as coupled design variables rather than isolated descriptors [5,6].

### 1.2. Multiscale Structural Design of OPV Bulk-Heterojunction Nanocomposites

The performance of OPV BHJ nanocomposites is strongly shaped by structural features spanning a wide range of length and time scales. At the sub-nanometre scale, the relative orientation and intermolecular spacing of donor and acceptor molecules at the D/A interface determine the electronic coupling that drives charge transfer [7,8]. At the 1–10 nm scale, local molecular packing—π-π stacking distance, crystallite size, and the degree of molecular order—controls exciton and charge transport mobility [9,10]. At the 10–100 nm scale, the phase-separated domain morphology—domain purity, interface width, and connectivity—strongly influences the balance between exciton harvesting and charge collection [11,12]. At the 100 nm-to-micrometre scale, thin-film interference effects, active-layer thickness, and electrode interfaces govern the optical field distribution and charge extraction efficiency [13,14].

No single experimental or computational technique can span this entire range. Instead, the rational design of OPV nanocomposites depends on a hierarchy of numerical modelling methods, each operating at its characteristic length scale, with information flowing between scales through carefully constructed interfaces. This multiscale modelling ecosystem includes quantum chemistry, atomistic and coarse-grained molecular dynamics, kinetic Monte Carlo, phase-field simulations, drift-diffusion device models, and increasingly, machine learning surrogates. The central challenge is not merely to apply these methods separately, but to pass physically meaningful parameters, uncertainties, and structure–property descriptors across scales.

### 1.3. From Fullerene to Non-Fullerene Acceptors: Expanded Structural Design Space

The transition from fullerene-based acceptors (e.g., PC61BM, PC71BM) to non-fullerene acceptors (NFAs), particularly the Y6 family of acceptor-donor–acceptor’-donor–acceptor (A-DA’D-A)-type small molecules, has expanded the structural design space of OPV BHJ nanocomposites [2,15,16,17]. Fullerene-based OPVs often allowed more reduced modelling descriptions because fullerene acceptors are relatively compact, electronically isotropic, and less structurally diverse than modern NFAs. In these systems, modelling efforts frequently centred on donor-polymer energetics, crystallinity, blend miscibility, and percolating transport pathways. NFAs, by contrast, often exhibit anisotropic molecular shapes, strong intramolecular charge-transfer character, and packing or crystallization behaviour that is more system-specific than in fullerene-based blends [18,19]. These features make morphology, interface structure, and device response more tightly coupled than in many fullerene-based blends.

Critically, many high-performing NFA systems also operate efficiently at small donor–acceptor energetic offsets, challenging simple descriptions based only on localized charge-transfer states or classical Marcus-type rate expressions [20,21]. The transport models considered in this review include kinetic Monte Carlo (KMC), delocalized kinetic Monte Carlo (dKMC), and jumping kinetic Monte Carlo (jKMC). In parallel, recent work has accelerated the adoption and further development of quantum-dynamical simulation methods—non-adiabatic molecular dynamics (NAMD) and exciton-state-based surface hopping (X-SH)—that explicitly treat the quantum-mechanical nature of exciton and charge dynamics [20,22,23].

### 1.4. Scope and Structure of This Review

**Figure 1:**
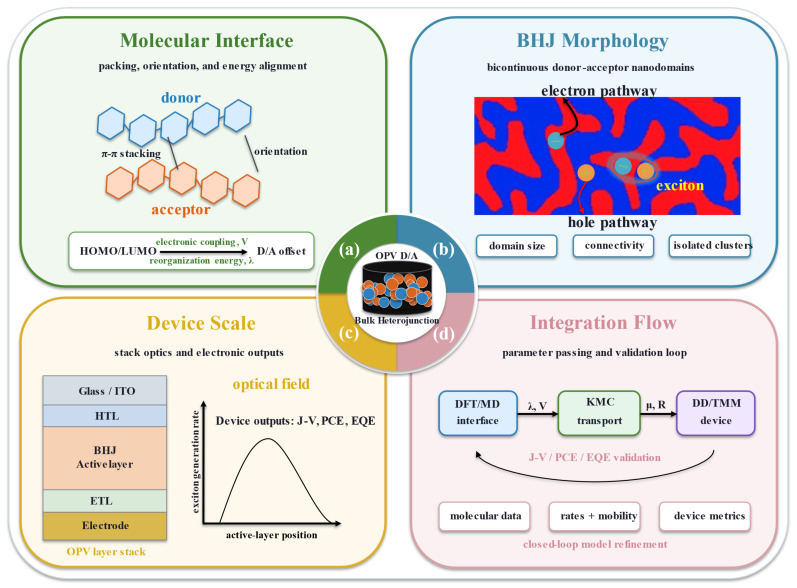
Multiscale structural hierarchy and cross-scale modelling workflow of OPV BHJ nanocomposites. (**a**) Molecular-interface structure, including donor–acceptor packing, molecular orientation, highest occupied molecular orbital (HOMO) and lowest unoccupied molecular orbital (LUMO) alignment, electronic coupling, reorganization energy, and D/A energetic offsets. (**b**) BHJ morphology at the mesoscale, where bicontinuous donor–acceptor nanodomains, domain size, phase purity, interfacial width, tortuosity, connectivity, and isolated clusters determine exciton harvesting and charge-transport pathways. (**c**) Device-scale response, linking the OPV layer stack—indium tin oxide (ITO), hole-transport layer (HTL), active layer, electron-transport layer (ETL), and electrode—and optical-field distribution to photovoltaic outputs such as current density-voltage (J-V) characteristics, external quantum efficiency (EQE), and PCE. (**d**) Cross-scale integration workflow, in which molecular and interfacial parameters from DFT/molecular dynamics (MD) calculations are transferred to kinetic Monte Carlo transport models and then to drift-diffusion (DD) and transfer-matrix method (TMM) device simulations, with J-V, PCE, and EQE validation feeding back into model refinement.

This review is organized around how structural information is generated, transferred, and validated across scales in OPV bulk-heterojunction nanocomposites. Figure 1 summarizes this organizing framework by linking molecular D/A interfaces, BHJ mesoscale morphology, device-scale optical and electrical response, and cross-scale parameter-passing with validation feedback. Its scope is donor/acceptor active layers in solution-processed OPVs, with particular emphasis on non-fullerene-acceptor-era blends; fullerene-based systems are discussed where they establish important modelling concepts or historical benchmarks. Section 2 examines molecular-scale descriptions of D/A interfaces, including density functional theory (DFT), time-dependent density functional theory (TD-DFT), non-adiabatic dynamics, and charge-transfer theories, with attention to energy-level alignment, electronic coupling, exciton/charge-transfer states, and descriptors that can be passed to higher-scale models. Section 3 discusses mesoscale morphology modelling through atomistic and coarse-grained molecular dynamics, kinetic Monte Carlo, electronic coarse-graining, and phase-field methods, focusing on domain size, phase purity, orientation, connectivity, processing history, and energetic disorder. Section 4 links these structural descriptors to optical generation, exciton and charge transport, recombination, and device-level performance through optical and drift-diffusion modelling. Section 5 and Section 6 treat machine learning and multiscale integration as cross-cutting tools: machine learning accelerates molecular screening, property prediction, and morphology optimization; whereas, integration frameworks define how parameters, uncertainties, and model boundaries are transferred between scales. Section 7 reviews experimental validation strategies and reporting requirements, and Section 8 summarizes persistent challenges and opportunities for cross-fertilization between OPV modelling and structural-composites thinking. Chemical synthesis routes, empirical processing recipes, and device architectures are not reviewed as standalone topics. Processing variables—including solvent and additive identity, blend ratio, evaporation history, annealing, and film thickness—are discussed only when they serve as model inputs, govern morphology evolution, or provide experimental constraints for model validation. This distinction separates processing chemistry from processing-aware numerical modelling. As this article is a review, no laboratory instrument or software package was used to generate new data; the methods and platforms named below are discussed as literature examples and are supported by the cited sources.

## 2. Molecular-Scale Modelling: D/A Interface Electronic Structure and Charge Generation

### 2.1. Density Functional Theory for Interfacial Electronic Structure

DFT remains a central tool for electronic-structure calculations in organic photovoltaics because it provides a quantitative starting point for the molecular frontier-orbital landscape. In donor–acceptor blends, and especially at the donor–acceptor (D/A) interface, HOMO and LUMO energies define the basic energetic offsets that underlie the thermodynamic driving force for charge transfer [24]. Functional choice is therefore central to whether these quantities are meaningful for interfacial states. Range-separated hybrid functionals such as ωB97X-D and CAM-B3LYP are widely used for charge-transfer excitation problems because self-interaction errors in semi-local and some conventional hybrid functionals can bias CT-state energetics [25]. Optimally tuned screened range-separated hybrid (OT-SRSH) approaches have also been used in perylene-diimide (PDI)-dimer NFA-related excited-state studies; broader claims about ionization-potential-theorem tuning and general prediction improvements for NFAs should remain source-dependent [26]. These molecular-interface descriptors correspond to the first level of the review framework shown in Figure 1a.

The application of DFT in OPV modelling has also moved from isolated molecular energy diagrams toward explicit D/A interface models. Bauch et al. combined molecular dynamics and DFT for interfaces formed by the polymer donor PM6 and the CF3-functionalized non-fullerene acceptor Y2CF3 (PM6:Y2CF3) interfaces and reported that CF3 functionalization narrows the singlet local-exciton (LE) to singlet charge-transfer (CT) gap, thereby promoting rapid LE-to-CT conversion [27]. These findings support faster LE-to-CT conversion; whereas, implications for open-circuit voltage should be evaluated separately and with appropriate system-specific caution [27]. Li et al. used (ab initio molecular dynamics) AIMD and DFT to compare the additive effects of 1,8-diiodooctane (DIO) and 3,5-dichlorobromobenzene (DCBB), assigning distinct microscopic mechanisms to the two additives: DCBB enhances localized directional charge transfer through hydrogen bonding; whereas, DIO improves packing and thereby promotes more delocalized transitions [28]. Together, these examples show how DFT-based workflows have evolved from static orbital pictures toward dynamic descriptions of interfacial chemistry.

High-throughput DFT screening extends this molecular-level picture into systematic structure–property exploration. End-group engineering across six NFAs identified AMF3 as a predicted candidate with open-circuit voltage = 1.262 V and (fill factor) FF = 90.20% [29]. Core-structure variation in six-fold rylene diimides has been used to compare how rylene-core and structural changes affect band gaps, HOMO/LUMO levels, optical absorption, and mobility, without implying that these effects are fully decoupled [30]. Side-chain engineering of PDI-core NFAs further suggests that flexible side chains are associated with prolonged charge persistence and delayed recombination, including a 6.59 ns recombination component, rather than a simple charge-separation lifetime assignment [31]. These examples establish DFT as a chemically interpretable source of molecular descriptors for screening, while also exposing a practical limitation: exhaustive DFT workflows rapidly become computational bottlenecks and therefore motivate the machine-learning acceleration discussed later in Section 5.

DFT provides the energetic blueprint, but not the dynamics. Frontier orbital energies, reorganization energies, interfacial couplings, and site energies are necessary descriptors, yet they do not by themselves reveal how excitations evolve after photoabsorption. The next step is therefore to move from ground-state electronic structure to explicit excited-state classification through TD-DFT.

### 2.2. TD-DFT and the Nature of Interfacial Excited States

TD-DFT extends the static DFT framework by calculating excited states and resolving their LE, CT, and hybrid character in explicit molecular examples [19]. More broadly, donor–acceptor-interface studies use LE/CT/hybrid-state language to interpret interfacial photophysics [32]. At a D/A interface, an LE state remains primarily localized on one molecular component, a CT state separates electron and hole density across the interface, and a hybrid state mixes these two limiting descriptions. This classification is not merely spectroscopic: the degree of LE/CT mixing determines whether a photoexcitation can access charge separation efficiently while avoiding excessive voltage loss. TD-DFT therefore supplies the state-resolved language needed to connect molecular packing, interfacial electronic structure, and device-level photophysics.

For TD-DFT descriptions of interfaces between the polymer donor PM6 and the non-fullerene acceptor Y6 (PM6:Y6), the main value is not a single optimized excited-state diagram but the ability to sample many disordered donor–acceptor complexes and separate Frenkel, CT, triplet, and non-radiative pathways [19]. The PM6:Y6 calculations summarized here indicate that Y6 excitations can contain intermolecular CT character, that triplet channels may either recycle through triplet-CT states or contribute to loss depending on energetic alignment, and that static disorder can accelerate CT-to-ground-state decay. The broader modelling implication is that excited states should be evaluated under finite-temperature interfacial disorder rather than only in optimized geometries.

The lowest charge-transfer energy (ECT) also anchors the voltage-loss discussion because it provides a fundamental upper limit for open-circuit voltage (VOC) [33]. A low non-radiative voltage loss of approximately 0.18 eV has been reported in a high-performing NFA system, but this value is source-specific and should not be generalized into a universal relationship between ECT and V_OC_ [34]. Reducing CT-state disorder may therefore lower non-radiative loss by narrowing the distribution of interfacial excited-state energies. This establishes a molecular-scale pathway used throughout the later sections: molecular packing influences CT-state character and disorder, CT-state properties affect charge-generation and recombination pathways, and those pathways ultimately shape device voltage losses. This molecular-scale discussion is also consistent with earlier photophysical work showing that charge photogeneration, CT-state relaxation, energetic driving force, and entropy can all contribute to the apparent ease of charge separation at D/A interfaces [35,36,37,38].

### 2.3. Non-Adiabatic Molecular Dynamics for Exciton Dissociation

DFT and TD-DFT provide static or state-resolved snapshots, but exciton dissociation itself occurs on femtosecond-to-picosecond timescales and is governed by strong electron-nuclear coupling. NAMD addresses this limitation by propagating electronic states and nuclear motion together. The relevant method family includes trajectory surface hopping (TSH), Ehrenfest molecular dynamics, and X-SH. Standard TSH is most naturally framed around stochastic transitions among localized electronic states; Ehrenfest MD treats electron-nuclear coupling in a mean-field manner; and X-SH is designed to propagate coupled excitonic and charge-transfer states at D/A interfaces. Their different approximations determine the balance between physical detail, coherence effects, system size, and accessible simulation time. Table 1 summarizes these method families and their main boundaries.

Surface-hopping NAMD extends the state-resolved picture by following how electronic character changes during nuclear motion [22]. In the X-SH picture discussed here, dissociation can be mediated by transiently delocalized excitonic (XT)-CT configurations rather than by a direct LE-to-CT jump; stronger electronic coupling or a higher dielectric environment shifts the dynamics from trapped cold configurations toward more effective hot pathways. The useful point for low-offset NFA systems is mechanistic: delocalized intermediate states can reduce the need for a large energetic driving force, but the claim should remain system-specific.

A complementary NAMD view emphasizes how the interface reshapes the initially local excitation before charge separation [23]. In the Y6-based example, the LE1 → LE2 → CT3 → CT1/CT2 sequence, enhanced exciton dipole moment, and lower reported CT-state binding energies relative to fullerene systems all point to the same conclusion: donor–acceptor contacts can weaken Coulomb binding through polarization and electronic coupling. The numerical offsets and binding energies are best treated as system-specific support for this mechanism rather than as transferable constants.

NAMD is also useful beyond the first interfacial dissociation event, because exciton harvesting depends on whether excitons can reach favourable interfaces before decay [40]. The crystal and thin-film results for the non-fullerene acceptor O-IDTBR cited here should therefore be used mainly as evidence for diffusion-length and disorder effects in their original structural context, not as a generic description of all NFA-rich phases. In this broader role, NAMD links molecular disorder to both exciton transport and interfacial charge generation.

### 2.4. Marcus Theory, Marcus–Levich–Jortner Theory, and Charge Transfer Rates

After DFT, TD-DFT, and NAMD define the relevant interfacial states and dynamical pathways, rate theories provide a compact language for comparing charge-transfer probabilities across molecular configurations. Ultrafast spectroscopic studies motivate this rate-based view because they show that delocalized or long-range charge-separated states may appear before complete thermal relaxation of the interfacial CT manifold [41,42,43].

Marcus theory supplies the classical rate framework that historically connected energetic driving force, reorganization energy, and donor–acceptor electronic coupling in OPV charge separation. In this framework, the electron-transfer rate depends on the donor–acceptor electronic coupling and the Franck–Condon weighted density of states, which is determined by the free-energy change and reorganization energy [44]. The inverted region captures the non-monotonic dependence of electron-transfer rate on driving force: beyond an optimal driving force, additional energy offset can reduce rather than increase the rate [44]. Historically, this framework explained why some energetic offset was considered necessary to separate Coulomb-bound excitons.

Marcus–Levich–Jortner (MLJ) theory is an important quantum-corrected extension for modern NFA blends, but its quantitative role should be treated as system-dependent. Recent Marcus-analysis discussions in NFA blends indicate that the comparison between classical Marcus and MLJ descriptions remains source- and assignment-dependent [45]. The supported MLJ correction is the inclusion of quantum tunnelling through high-frequency vibrational modes, without assigning a universal role to any specific vibrational mode [45]. Iwasaki et al. further support the concept that minimal driving force can enhance electron transfer when the reorganization energy is small, although this evidence comes from an organic-semiconductor interface study rather than a direct NFA OPV proof [21]. Thus, the NFA-era interpretation of “driving force” should remain coupled to molecular rigidity and quantum nuclear motion without overextending a single citation.

Wave-packet dynamics provide another route beyond the single-step Marcus picture. Fujita and Nozaki showed that the wave-packet dynamics in which higher CT-state participation can facilitate exciton dissociation, although the explicit LE → CT1 → CT2 → free ladder should be treated cautiously [46]. Such multistate pathways may lower the effective kinetic barrier relative to a single-step Marcus process. However, all of these theories still retain a common assumption: the relevant charge carriers are localized on molecular sites. The next methodological step is to relax that assumption, because delocalization is no longer a negligible perturbation in many high-performing NFA systems.

### 2.5. Beyond Marcus: Delocalization and Quantum Coherence

Delocalization-based transport models challenge the assumption that charge generation must always proceed through nearest-neighbour localized hopping [20]. In dKMC, explicit treatment of disorder, polaron formation, and quantum delocalization predict that longer-range transitions can bypass tightly bound CT configurations and may help maintain efficient generation even at small, and in source-specific cases near-zero, energetic offsets. For OPV design, the implication is to treat energetic offset and electronic coupling as coupled variables: close π-π packing, extended conjugation, and backbone rigidity may be as important as the nominal donor–acceptor energy difference.

Lower-cost delocalization-corrected KMC methods occupy the practical middle ground between fully delocalized simulations and classical hopping models [47]. jKMC retains the site-based KMC framework but adds corrections for partial wavefunction extension, allowing delocalization effects to be estimated at costs closer to ordinary KMC. The reported mobility enhancement illustrates the possible scale of the effect, but its magnitude should be read as model-specific; the more general lesson is that the localized-hopping approximation should be tested whenever intermolecular coupling is strong.

**Figure 2:**
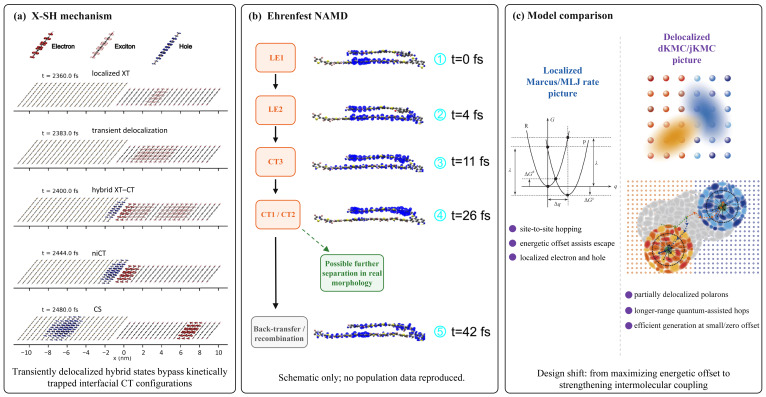
Conceptual synthesis of non-adiabatic and delocalized charge-generation mechanisms at donor–acceptor interfaces. (**a**) X-SH mechanism in which localized XT configurations transiently access delocalized hybrid XT-CT states before evolving toward non-interfacial charge-transfer (niCT) and charge-separated (CS) states, thereby bypassing kinetically trapped interfacial CT configurations. (Reproduced from Ref. [22], distributed under the terms of the Creative Commons Attribution 4.0 International Licence (CC BY 4.0)). (**b**) Schematic Ehrenfest NAMD charge-transfer sequence for a Y6-based donor–acceptor interface, illustrating the reported progression from local exciton states (LE1, LE2) to charge-transfer states (CT1/CT2/CT3), followed either by further separation in a realistic morphology or by back-transfer/recombination. The time labels and molecular snapshots are illustrative state-sequence markers and do not reproduce quantitative population dynamics. (Adopted from Ref. [23]). (**c**) Comparison between localized Marcus/MLJ-type descriptions and delocalized dKMC/jKMC-type models. In the localized picture, charge generation is described mainly through site-to-site hopping, energetic offsets, reorganization energy, and localized electron-hole separation. In the delocalized picture, partial polaron delocalization and longer-range quantum-assisted hops can support efficient charge generation even at small or near-zero energetic offsets. (Adopted from Ref. [20]). Together, the three panels illustrate a design shift from relying primarily on energetic offset toward strengthening intermolecular coupling, controlling disorder, and enabling delocalized charge-transfer pathways.

The conceptual shift can therefore be summarized as a move from an energy-offset-only picture toward a framework that also emphasizes coupling, delocalization, and disorder. These views are not mutually exclusive but emphasize different limiting descriptions. In the classical view, larger offsets help overcome Coulomb binding at the cost of voltage loss. In the quantum view, strong intermolecular interactions and partial delocalization can provide a route to efficient charge generation without imposing a large energetic penalty; the OPV-specific synthesis relies most directly on [20,47]; whereas, the minimal-driving-force concept is supported more generally by [21]. This perspective also rationalizes structural features emphasized across the cited examples, including planar and rigid cores, strong intermolecular interactions, and packing motifs that support electronic coupling [3,48,49]. Figure 2c summarizes this localized-to-delocalized model shift by comparing Marcus/MLJ-type rate descriptions with dKMC/jKMC-type pictures.

### 2.6. Molecular-Scale Modelling Summary

Molecular-scale modelling of OPV interfaces has progressed from static DFT energy diagrams to increasingly dynamic and quantum-mechanical descriptions. DFT defines the energetic blueprint through HOMO/LUMO offsets, reorganization energies, and electronic couplings [25,26,27,28,29,30,31,32]; whereas, TD-DFT resolves LE, CT, and hybrid excited states and links excited-state character to charge-generation rates, CT-voltage relations, and non-radiative loss channels [22,35,36,37]. NAMD then adds real-time electron-nuclear dynamics, including hybrid XT-CT states [22] and polarized local-exciton pathways [23], while Marcus-type descriptions may require MLJ or wave-packet corrections when high-frequency vibrations and partial delocalization are important [45,46]. Taken together, these methods show that delocalization should be treated as a design variable coupled to electronic coupling, energetic offset, morphology, and disorder rather than as a secondary correction [23,24,49].

These methods are complementary rather than interchangeable. DFT and TD-DFT provide electronic descriptors and excited-state classifications; whereas, NAMD offers direct access to ultrafast electron-nuclear dynamics at substantially higher computational cost and with more limited sampling [19,22,23,24,25,26,39,40]. Marcus/MLJ and KMC-type descriptions extend accessible time and length scales, but their conclusions depend on localization assumptions, rate-law selection, and disorder parameterization [20,44,45,46,47]. Accordingly, mechanistic claims are strongest when static energetics, dynamical simulations, and independent spectroscopic or transport measurements are mutually consistent.

## 3. Mesoscale Morphology Modelling

### 3.1. The Morphology Challenge: From Non-Equilibrium Film Formation to Computable Morphology

A BHJ is not an equilibrium two-phase mixture. During coating, solvent evaporation, liquid–liquid demixing, nucleation, crystallization, and vitrification occur over overlapping time windows, leaving a morphology that is arrested kinetically. Nor can a useful morphology be reduced to one length scale. Domains that are too large impede exciton access to an interface; whereas, excessively fine mixing can lower phase purity and create trap-rich intermixed regions. Interfacial area, interfacial width, molecular orientation, phase purity, and connectivity must therefore be considered together; “finer phase separation” alone is not a sufficient design rule [50]. These coupled morphology descriptors are summarized schematically in Figure 1b.

The useful product of a mesoscale model is a set of transferable, testable descriptors. Radial distribution functions, orientation distributions, domain dimensions, interfacial widths, and structure factors can be compared with structural measurements. Site energies, energetic disorder, electronic couplings, mobilities, and recombination parameters provide a route to transport and device models. A single representative snapshot is often insufficient for either purpose: it hides sample-to-sample variation and gives no indication of how strongly a device calculation depends on morphology selection. Morphology distributions and their uncertainty are more informative, provided that each output is tied to a specific downstream model.

### 3.2. All-Atom and Coarse-Grained Molecular Dynamics

All-atom molecular dynamics (AA-MD) retains bonding, side-chain conformations, and local electrostatics, which makes it appropriate for π-π contacts, donor–acceptor motifs, and interfacial orientation. Sampling is the principal limitation: nanosecond trajectories and finite boxes seldom encompass complete film formation or domain coarsening. Coarse-grained molecular dynamics (CG-MD) reaches larger boxes and longer times, but smooths the energy landscape, accelerates dynamics, and removes direct access to electronic structure. Whether a CG representation is adequate consequently depends on the intended observable, rather than on density or visual resemblance alone.

Two benchmark studies illustrate this dependence on purpose. Roscioni and co-workers compared structure, carrier energy levels, energetic disorder, and intermolecular coupling in amorphous organic semiconductors. A carefully parameterised anisotropic CG model approached the atomistic reference, while backmapped samples gave electronic properties that were quantitatively consistent with native atomistic structures [51]. Wang and co-workers tested a different requirement: thermomechanical consistency. Their energy-renormalisation model reproduced density, Debye-Waller factors, Young’s modulus, and trends in glass transition across PDPPT architectures [52]. Backmapping may recover local electronic environments, but processing and stability questions also require temperature, modulus, and relaxation to be checked.

Atomistic and coarse-grained MD show why BHJ morphology cannot be reduced to average domain size [4]. In PM6:Y6, coarse-grained simulations indicate that interfacial width, acceptor connectivity, donor–acceptor contact chemistry, and isolated acceptor clusters can vary independently. The morphology problem is therefore not a single size-optimization problem: percolation, local electronic complementarity, and phase purity must be evaluated together.

Consistent with Section 1.4, additives are discussed here not as empirical processing recipes, but as modelled perturbations to packing, mixing, and interfacial partitioning [12]. The PM6:PYIT system, comprising the polymer donor PM6 and the polymer acceptor PYIT, shows that closely related solvent additives can strengthen donor π-π packing, promote donor–acceptor separation, or weaken these effects through steric substitution. The general point is that an additive can modify donor aggregation, blend thermodynamics, and interface structure in different directions, so its effect should not be inferred from one morphology metric alone. The practical distinctions among mesoscale methods are summarized in Table 2.

### 3.3. Electronic Coarse-Graining and Morphology-to-Electronic Mapping

ECG is best understood as a morphology-to-Hamiltonian acceleration strategy rather than a replacement for atomistic electronic-structure data [53]. A small atomistic training set can be used to learn mappings from CG geometry to site energies and electronic couplings, allowing large morphology ensembles to be evaluated at CG resolution. The reported benzothieno [3,2-b]benzothiophene (BTBT) application demonstrates the possible cost reduction, but reliability in chemically heterogeneous OPV blends still depends on training coverage, interfacial extrapolation, and out-of-distribution detection.

The morphology section also benefits from broader OPV morphology literature: processing, crystallization, domain purity, and characterization protocol determine whether simulated structures are physically comparable with measured films [54,55,56,57].

### 3.4. Kinetic Monte Carlo for Exciton and Charge Transport

KMC performs a different reduction. It converts a morphology into a network of sites and propagates excitons or charges with Marcus, Marcus–Levich–Jortner, or related rates. Mobilities, diffusion lengths, generation yields, and recombination coefficients can then be estimated, but these quantities are not functions of geometry alone. Site definitions, the mapping of disorder and couplings, and device boundary conditions all affect the result [58]. KMC is best viewed as a morphology-to-transport bridge, not as a transport predictor detached from the morphology model that supplied its inputs.

Excited-state KMC networks can carry more physics than a simple exciton-to-free-carrier yield parameter [19]. The PM6:Y6 network discussed earlier connects Frenkel excitons, CT states, triplets, static disorder, and dynamic disorder in a single transport scheme. Its main contribution to this section is not another PM6:Y6 case study, but a caution for KMC parameterization: triplet states may recycle or become losses depending on the energy landscape, so their role should not be hard-coded as universally terminal.

The localized-hopping assumption also warrants testing in strongly coupled NFAs. dKMC, which treats disorder, polaron formation, and partial delocalisation explicitly, showed that longer-range transitions can facilitate charge generation [20]. jKMC incorporates a delocalisation radius into a correction to the Marcus rate and approaches dKMC accuracy at a cost close to ordinary hopping KMC; calculated mobilities increased by as much as two orders of magnitude [47]. These methods provide a hierarchy rather than a universal upgrade. Once a wavefunction extends over distances comparable with intermolecular spacing, the bias introduced by strictly local hopping should at least be quantified.

A joint calculation-experiment study of O-IDTBR provides a stringent example. In the ideal crystal, the excess electron extended over about three molecules and the calculated high-mobility direction reached approximately 7 cm^2^ V^−1^ s^−1^. Static off-diagonal disorder in thin-film structures then converted transport to small-polaron hopping. The larger reduction came from diagonal electrostatic disorder associated with the A-D-A architecture, which lowered mobility by a further four to five orders of magnitude to about 10^−5^ cm^2^ V^−1^ s^−1^, consistent with space-charge-limited-current (SCLC) estimates [9]. Geometric connectivity is necessary, but the spatial electrostatic landscape may dominate transport in the film.

### 3.5. Phase-Field Modelling and Processing History

Phase-field models exchange molecular contacts for continuous variables such as local composition, crystallinity, and orientation. Cahn–Hilliard equations describe conserved composition fields, while Allen–Cahn-type equations can represent non-conserved crystallization variables. This loss of microscopic detail makes hundred-nanometre and long-time processing simulations possible. In the coupled model of Ronsin and Harting, nuclei could initiate spinodal decomposition; whereas, growing crystals arrested coarsening in the amorphous mixture; pre-existing composition-rich regions in turn guided crystal growth [59]. The outcome is a mechanism map, not a transferable processing recipe, because quantitative predictions still require independently measured thermodynamic and kinetic parameters.

For KMC-based morphology-to-transport mapping, earlier mesoscopic and methodological studies remain relevant because electrostatic interactions, dimensionality, and lattice/coarse-graining assumptions can alter simulated charge-collection trends [60,61,62].

Numerical tractability and physical calibration solve different problems. Bergermann and co-workers developed Schur-complement preconditioning for a ternary donor–acceptor-solvent Cahn–Hilliard model, making the finite-element system robust to changes in mesh and numerical parameters. Their proof-of-concept device calculation also showed that morphology resolution affected J-V curves and charge density [63]. Ameslon and co-workers instead compared phase-field simulations directly with experimental morphologies of the DRCN5T:PC71BM blend during annealing, where DRCN5T is a small-molecule donor and PC71BM is a fullerene acceptor. The evolution was governed mainly by dissolution of the smallest unstable crystals and anisotropic growth of the largest stable crystals, rather than simple mean-grain Ostwald ripening [11]. One study addresses whether the equations can be solved efficiently; the other asks which mechanism is consistent with the experiment.

Coupled phase-field/device models illustrate how processing history can be passed into device-scale observables without retaining molecular detail [64]. A finite-element workflow can link phase-field morphology, exciton dynamics, and drift-diffusion variables such as carrier densities, potential, and J-V response. At present, such models are better framed as extensible coupling architectures than as complete OPV device descriptions, because thermal effects and trap-related energetic disorder still need independent treatment.

### 3.6. Cross-Scale Information Transfer and Section Summary

A defensible chain from processing to device response can be stated explicitly: processing conditions generate a distribution of morphologies; those morphologies define local energies and couplings; transport models compress the statistics into mobilities, diffusion lengths, and recombination parameters. Each compression should preserve the quantities required at the next scale. A device model with density-dependent mobility, for example, cannot be supported by a single zero-field, single-carrier mobility. Likewise, experimentally observed vertical segregation cannot be represented by a laterally averaged two-dimensional morphology without assessing the resulting bias.

Uncertainty propagation remains the weakest part of this chain. Force fields, mapping choices, quantum-chemical methods, and finite-size effects all introduce error, yet those errors are commonly hidden when one deterministic number is passed to the next model. Repeating calculations over independent morphologies, reporting descriptor distributions, and testing whether device conclusions remain stable across those distributions would be more useful than enlarging one simulation box indefinitely. The aim is to separate a material trend from the accidental features of a selected trajectory.

A materials-design interpretation also requires controllable variables to be connected directly to model descriptors. Solvent choice and evaporation rate primarily alter mixing, nucleation sequence, and vertical segregation; annealing changes crystal stability and coarsening; backbone, end-group, and side-chain chemistry jointly affect conformation, compatibility, and electrostatic disorder. A model that cannot identify which processing or molecular variable controls a descriptor may explain a morphology, but it does not yet guide materials design. Table 3 places common control variables, structural responses, properties, and validation methods in one matrix. The experimental techniques used in Table 3 include resonant soft X-ray scattering (RSoXS), grazing-incidence wide-angle X-ray scattering (GIWAXS), atomic force microscopy (AFM), transmission electron microscopy (TEM), ultraviolet photoelectron spectroscopy (UPS), and inverse photoelectron spectroscopy (IPES); DOS denotes density of states.

## 4. Device-Scale Modelling

### 4.1. Optical Modelling: Source Terms and Model Boundaries

The optical problem in a planar multilayer OPV is usually treated with TMM. Complex refractive indices and layer thicknesses determine forward and backward waves, from which |E(x, λ)|^2^, layer-resolved absorption, and the exciton-generation profile G(x, λ) follow. The early model of Pettersson and co-workers combined thin-film interference with exciton diffusion and interfacial quenching, obtaining diffusion-length ranges of 4.7 and 7.7 nm and showing that optical generation and exciton collection cannot be treated as unrelated steps [65]. A generation profile used in drift-diffusion should therefore come from an optical model that has first been tested against absorption or EQE. This device-scale link between layer structure, optical generation, and photovoltaic outputs is represented in Figure 1c.

TMM-Sim brings electric-field profiles, generation, layer absorption, parasitic loss, EQE/internal quantum efficiency (IQE), and photocurrent into one tool and exports generation profiles for electrical simulation. In its bilayer example, thickness optimization increased the calculated photocurrent from 4.02 to 6.89 mA cm^−2^, a rise of about 58% [13]. This is an optical result under specified constants and collection assumptions, not evidence for an equal increase in measured PCE. Its most defensible uses are layer-thickness screening and localisation of parasitic absorption.

Lateral textures, nanoparticles, and gratings invalidate the one-dimensional layered assumption and require the finite-difference time-domain (FDTD) method or another full-wave method. A double-ridge FDTD model produced a tunable absorption feature near 530 nm whose position depended on both the ZnO and active-layer ridges [66]. A separate study of Ag parabolic arrays predicted increases of 26.41% in short-circuit current and 26.37% in PCE relative to a planar reference [67]. Such numbers are optical-design upper bounds until metal absorption, morphology disturbance, fabrication tolerance, and charge collection are included.

### 4.2. Drift-Diffusion Modelling: Disorder and Parameter Identifiability

A DD model describes charge transport at the continuum device scale. In its minimal form, it couples the electrostatic potential with the conservation of electron and hole populations, so that photogeneration, carrier motion, recombination, and charge extraction at contacts are treated within a single self-consistent framework. The value of the DD approach is therefore not that it resolves every microscopic process, but that it connects these processes to measurable device-level behaviour. For organic semiconductors, the predictive accuracy of a DD simulation often depends less on the formal structure of the model than on the physical assumptions used to close it. These include the density-of-states description, field- or density-dependent mobility, trapping and detrapping, recombination pathways, and contact boundary conditions. A reproducible DD study should therefore report not only that a DD model was used, but also the constitutive relations, material parameters, and boundary conditions adopted in the simulation.

Drift-diffusion models are most useful when they separate generation loss from post-generation transport and recombination loss [68]. In the PPV:PCBM example, field- and temperature-dependent generation and Langevin recombination were fitted within one device model, giving system-specific estimates for bound-pair dissociation and subsequent bimolecular loss. The transferable lesson is methodological: generation efficiency, mobility, and recombination should be constrained separately rather than absorbed into a single effective efficiency parameter.

Disordered organic layers cannot always be represented by Boltzmann statistics and a constant mobility. Clarke and co-workers showed that non-Boltzmann statistics in highly doped organic transport layers materially changed predicted open-circuit voltage and PCE [69]. Coehoorn and Bobbert derived compact temperature-, field-, and density-dependent mobility expressions from three-dimensional Gaussian-DOS hopping simulations: uncorrelated disorder gives the extended Gaussian disorder model (EGDM); whereas, dipolar correlated disorder gives the extended correlated disorder model (ECDM) [70]. A one-dimensional DD calculation may still reproduce average current when microscopic pathways are filamentary, but only if the mobility law is consistent with the assumed disorder.

Device simulations should generally be interpreted as parameterized trend analyses unless their inputs are independently constrained [14]. The OghmaNano thickness/interlayer scan illustrates this point: a model can report a precise optimum PCE and current density, but those values are not certified device measurements. Without measured optical constants, mobilities, recombination rates, and contact parameters, several parameter sets can reproduce similar J-V curves; the simulation is then better suited to ranking trends than to making absolute performance claims.

Apparent recombination order can reflect the assumed density of states and trapping model rather than a new elementary recombination reaction [71]. The P3HT:PCBM moving-boundary treatment showed that free and trapped populations, density-dependent mobility, and an exponential DOS tail can reproduce measurements that a purely Gaussian DOS cannot. This example supports a broader fitting rule: recombination interpretation is inseparable from how energetic disorder and mobility are represented.

Identifiability improves when drift-diffusion parameters are constrained by several independent observables [72]. Thickness-dependent photocurrent, optical modelling, illuminated J-V curves, and turn-off photocurrent transients each restrict different parts of the model, from photon-to-CT conversion to CT dissociation and carrier mobility. This multi-observable strategy is more informative than fitting a single J-V curve because it reduces compensation among optical, generation, transport, and recombination parameters. Table 4 links the principal DD inputs to independent observables and common identifiability risks. The validation methods in Table 4 include transient photovoltage (TPV) and charge extraction (CE).

### 4.3. Energy- and Power-Loss Analysis

The open-circuit voltage cannot be reduced to the HOMO-LUMO difference in two isolated materials. Vandewal and co-workers connected CT absorption, CT emission, and detailed balance to show that open-circuit voltage in polymer-fullerene cells is governed by interfacial CT states and their radiative and non-radiative recombination [33]. Low-energy EQE, electroluminescence efficiency, and dark saturation current are thereby placed in the same thermodynamic framework, providing an experimental route to non-radiative voltage loss.

NFA devices require the CT-state disorder, back charge transfer, and triplet pathway to be separated further. In D18:N3-BO:F-BTA3, a ternary blend comprising the polymer donor D18, the host non-fullerene acceptor N3-BO, and the guest acceptor F-BTA3, tighter packing reduced CT-state disorder slowed back charge transfer and suppressed triplet formation; the device reached ΔV_nr_ = 0.183 eV and PCE = 20.25% [34]. The result supports a morphology-CT landscape-non-radiative recombination-open-circuit-voltage chain, but it remains evidence from one ternary system. Low CT disorder is not, by itself, a sufficient condition for every donor–acceptor pair.

Voltage accounting captures only part of the loss. A power-loss model can also assign thermalization, recombination, CT-exciton dissociation, and interfacial energetic offsets to separate power terms. In the examples studied by Mehdizadeh-Rad and co-workers, photocarrier flux and recombination lifetime dominated output; whereas, the importance of energetic offsets and CT dissociation depended on the parameter regime [73]. The decomposition is useful for comparison only when each term is constrained by measurable quantities; otherwise, the same total loss can be redistributed among poorly identified components.

Device-scale parameterization should also account for established optical and charge-transport corrections, including interference/scattering corrections in optical measurements, density- and field-dependent mobilities, and transient photocurrent constraints [74,75,76,77,78].

### 4.4. Simulation Platforms and Model Complexity

Platform choice should follow the question. TMM-Sim is suited to planar optical fields and G(x); OghmaNano, the successor name to gpvdm, includes DOS, recombination, and transient models; SETFOS supports commercial optoelectronic fitting. SIMsalabim provides an open-source one-dimensional DD implementation for reproducible batch studies [79]. SolarDesign places material, device, and circuit levels in an online multiphysics environment and includes specialized processes such as tunnelling, exciton dissociation, and ion migration [80]. More available physics does not by itself strengthen an inference. Inputs and assumptions remain the limiting factors. The scope of each platform is compared in Table 5.

For a practical modelling study, the simplest identifiable model is usually the stronger starting point. Measured n, k, and thickness first define the optical source term. Mobilities, recombination, and contacts are then added only when independent data constrain them. Tail states, field-dependent mobility, interfacial barriers, or transient modules should enter in response to a specific residual. FDTD is justified when lateral structure breaks the planar approximation; TPV, impedance, or transient current becomes necessary when steady-state measurements cannot distinguish parameters. Evidence, rather than available software features, should set model complexity.

### 4.5. Vertical Morphology, Interlayers, and Contact Selectivity

One-dimensional device models commonly treat the active layer as laterally homogeneous with depth-independent parameters; whereas, solution-processed films may contain donor–acceptor concentration gradients, surface enrichment, and electrode-proximal differences in crystallinity. These features do more than change an average mobility. They alter local absorption, the internal field, carrier accumulation, and interfacial recombination. A mesoscale output limited to a bulk-average phase fraction cannot distinguish bulk transport limitation from extraction loss near a contact.

The role of an interlayer likewise cannot be reduced to a work-function shift. Selectivity, interface dipoles, defect passivation, surface energy, and processing-induced vertical composition may change together. The interlayer scans of Boudia and Zhao demonstrate the sensitivity of device predictions to contact configuration and active-layer thickness [14], while the thickness series and transient analysis of Häusermann and co-workers provide a route to separating bulk and contact effects [72]. A materials-level model should treat cross-sectional composition, interfacial energetics, and contact recombination velocity as distinct inputs rather than allowing bulk mobility to absorb all interfacial error.

Voltage-loss analysis should therefore distinguish energetic offsets, disorder, exciton lifetime, and radiative versus non-radiative recombination channels, rather than assigning voltage loss to a single descriptor [81,82].

Device-scale simulation is therefore not a single-step conversion from material parameters to efficiency. A defensible chain first obtains G(x) from an experimentally tested optical model, then solves carrier distributions with transport and recombination laws consistent with the assumed disorder, and finally interprets losses in J-V, EQE, open-circuit voltage, and fill factor through contacts and vertical morphology. The provenance, range, and residual associated with each input should remain visible at every stage. Otherwise, downstream fitting can compensate for upstream error and produce an accurate device curve with little materials-level meaning.

## 5. Machine Learning for Accelerated Structural Design

### 5.1. The Data-Driven Transformation

Machine learning (ML) has become a necessary complement to first-principles, molecular, mesoscale, and device-level modelling because the design space of OPV donor–acceptor composites grows combinatorially. Thousands of possible donors can be paired with thousands of acceptors, and each molecular pair must still be evaluated across blend ratios, additives, annealing histories, film thicknesses, and device architectures. Exhaustive trial-and-error exploration is therefore impractical even before multiscale structural variables are considered [83]. Within this landscape, ML operates across several levels of the modelling hierarchy: molecular models predict electronic and photovoltaic properties, mesoscale models accelerate force-field development and morphology descriptor extraction, device models learn links to J-V response, and design algorithms combine generative modelling with active learning. Within the framing of this review, ML is therefore treated as an increasingly important component of multiscale OPV modelling rather than as a replacement for physical simulation [83].

**Figure 3:**
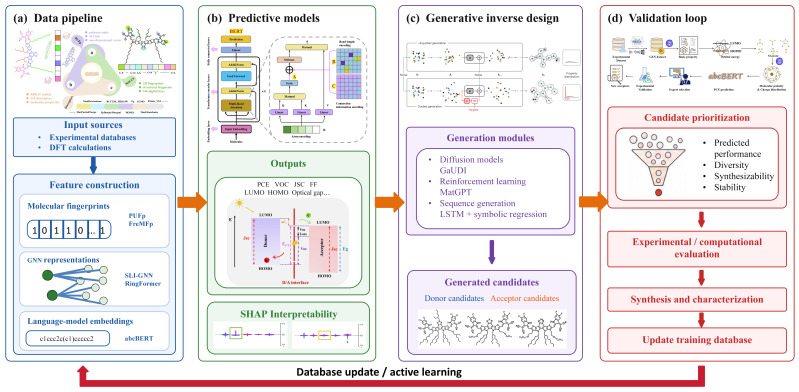
Machine-learning workflow for accelerated structural design of OPV donor–acceptor nanocomposites. (**a**) Data pipeline in which experimental databases and quantum-chemical calculations provide input structures and properties for feature construction, including molecular fingerprints such as the polymer unit fingerprint (PUFp) and frequency molecular fingerprint (FreMFp), graph neural network (GNN) representations such as the self-learning-input GNN (SLI-GNN) and RingFormer, and language-model embeddings such as abcBERT. (Reproduced from Ref. [84], distributed under the terms of the Creative Commons Attribution 4.0 International Licence (CC BY 4.0)). (**b**) Predictive models that map these representations to photovoltaic and molecular-property outputs, including PCE, VOC, short-circuit current density (JSC), FF, HOMO/LUMO levels, and optical gaps; interpretability tools such as Shapley additive explanations (SHAP) analysis can further identify correlation-informed structural or processing descriptors. (Reproduced from Ref. [85], distributed under the terms of the Creative Commons Attribution 4.0 International Licence (CC BY 4.0)). (**c**) Generative inverse-design module, where diffusion models, reinforcement-learning strategies, and sequence-generation approaches such as GaUDI, MatGPT, and long short-term memory (LSTM)-symbolic-regression workflows propose or prioritize new donor and acceptor candidates under target-property constraints. (Reproduced with permission from Ref. [86], copyright 2023 Springer Nature). (**d**) Validation and active-learning loop in which predicted candidates are filtered by performance, diversity, synthesizability, and stability, then evaluated through higher-level computation, synthesis, characterization, or device testing. The resulting data are fed back into the training database, closing the loop between prediction, generation, validation, and model updating. Blue, green, purple, and red borders group the data, prediction, generation, and validation stages, respectively; orange arrows indicate forward information flow, and the red return arrow denotes database updating and active learning.

This section follows the conceptual progression required for data-driven structural design. Section 5.2 first considers forward property prediction, where the central question is how molecular structure can be translated into descriptors, learned representations, and photovoltaic parameters. Section 5.3 then turns to generative inverse design, where the task shifts from predicting known candidates to proposing new molecular structures. Section 5.4 examines high-throughput screening and active learning as routes for prioritizing experiments and computations. Finally, Section 5.5 addresses interpretability, where ML outputs are connected back to physically meaningful design hypotheses and to the scale-bridging workflows developed in Section 6. Figure 3 summarizes this data-driven workflow, from data and representation construction to property prediction, generative inverse design, candidate prioritization, and database-updating active learning.

### 5.2. Property Prediction: From Molecular Structure to Key Parameters

Descriptor-based ML remains valuable for sparse OPV datasets because experimentally accessible molecular descriptors can encode part of the structure-performance relationship [87]. The Y6-based descriptor/gradient-boosting study illustrates this early regime: useful PCE predictions could be obtained without directly using HOMO or LUMO levels as input descriptors. The limitation is structural rather than purely statistical: fixed descriptors and fingerprints are not optimized for the prediction task and may miss NFA-specific motifs that control packing, miscibility, and interfacial energetics.

Learned and task-adapted fingerprints attempt to close this gap by encoding OPV-relevant molecular fragments more directly. The PUFp approach used a Python-based polymer-unit-recognition script (PURS), as reported in the cited study, to construct a 413-bit representation for 1343 experimentally validated NFA materials, then paired this representation with a random forest (RF) model and outperformed Molecular ACCess System (MACCS) fingerprints [84]. The same workflow generated 3336 virtual acceptor combinations for screening, linking representation design to candidate discovery [84]. A related direction is the FreMFp, which was combined with CatBoost to screen a 6-million-donor space and reached a reported R^2^ of 0.82, including prediction of a D18-D2Cl:L8-BO candidate, comprising the screened polymer donor D18-D2Cl and the non-fullerene acceptor L8-BO, with a PCE of 19.59% [88]. These examples show that fingerprints are no longer merely generic encodings; they increasingly function as chemistry-aware filters for large OPV design spaces.

GNNs extend this trend by allowing molecular graphs to define the representation rather than relying entirely on preselected descriptor vectors. The SLI-GNN plus Light Gradient Boosting Machine (LightGBM) framework reached a reported Pearson correlation coefficient of r = 0.79 and gave experimentally validated errors below 2%, indicating that graph-derived structural information can be useful beyond retrospective fitting [89]. RingFormer further reflects the structural specificity of current OPV materials: by representing three graph levels, from atoms to rings to inter-ring connections, it targets ring-rich OPV molecular structures and reports a 22.77% relative improvement on the benchmark considered in the source [90]. DeepDonor used quantum-descriptor fingerprints for donor discovery, with reported correlations of r = 0.82 for small molecules and r = 0.77 for polymers, and was also made available as an online tool [91]. The residual-gated graph neural network (RGNN), trained on 48 K TD-DFT data, reported HOMO/LUMO R^2^ values of approximately 0.84–0.85 [92].

Transformer-based models extend OPV property prediction by separating broad chemical pretraining from smaller experimental performance datasets [85]. DeepAcceptor is useful as an example of this strategy because DFT-scale pretraining is combined with finetuning on reported NFA PCE data, while benchmark HOMO/LUMO errors remain dataset-specific rather than generally equivalent to DFT accuracy. The methodological message is that representation learning can transfer information from large calculated corpora to sparse OPV measurements, but the target-domain validation still governs reliability.

The progression from Dragon descriptors to task-specific fingerprints, graph neural networks, and transformers therefore represents a shift from manually fixed molecular summaries to self-learning representations. This shift matters because OPV structure–property relationships are not controlled by isolated scalar inputs alone, but by interacting molecular fragments, packing tendencies, electronic levels, and processing-sensitive device contexts. Forward prediction is nevertheless only the first stage of accelerated design; once the mapping from structure to property becomes reliable enough, the next question is how to invert that mapping and propose new molecular structures. Table 6 summarizes representative ML property-prediction approaches discussed above. Table 6 uses gradient-boosted regression trees (GBRT), mean absolute error (MAE), and the simplified molecular-input line-entry system (SMILES).

### 5.3. Generative Models for Inverse Molecular Design

The OPV-specific data-driven literature also connects to broader high-throughput materials discovery: the Harvard Clean Energy Project established large-scale virtual screening as a practical materials-discovery paradigm, while later neural-network work showed how learned surrogates can accelerate screening across chemical spaces [94,95].

Generative modelling changes the central question of ML-assisted OPV design. Forward prediction asks what PCE or electronic property should be expected from a proposed donor–acceptor candidate; inverse design asks what molecular structure would maximize a target property under the constraints of valid chemistry and device relevance (Figure 3c). Four major generative routes are relevant to this discussion: variational autoencoders, generative adversarial networks, diffusion models, and reinforcement learning. These approaches are conceptually important because they move ML beyond ranking existing candidates and toward the construction of new chemical hypotheses that can later be filtered by physics-based simulation, high-throughput screening, or experiment.

General molecular diffusion models are relevant to OPV inverse design mainly as methodological benchmarks rather than direct OPV workflows [86]. GaUDI shows how equivariant property prediction and denoising diffusion can maintain high molecular validity, a necessary condition for exploring specialized NFA-like chemical spaces. However, chemical validity alone does not establish OPV usefulness; generated structures still need screening for absorption, energy alignment, packing, miscibility, processability, and device compatibility.

OPV-specific generative workflows connect molecular generation with screening at very large scale. Lv et al. used character-level LSTM generation across 210,660 donors and 878,268 acceptors, and combined the workflow with symbolic regression through gplearn to identify PCE-relevant descriptors [96]. The cited work reports a strong positive association between bicyclic fragment counts and PCE, as well as 5753 donor–acceptor pairs with predicted PCE above 18.5% [96]. CycleChemist is described as a two-pronged platform integrating OPVC, MOE2, P3, and MatGPT with reinforcement learning, indicating that modern OPV discovery workflows may combine predictive, generative, and evaluation modules rather than selecting a single algorithmic family [97]. This integration begins to blur the boundary between generative design and high-throughput prioritization.

Score-based and multi-granularity generators point toward broader chemical exploration, but their OPV value depends on downstream physical screening [98]. OrgMol-Design should therefore be cited as methodological support for molecular generation rather than as a demonstrated OPV design pipeline. For OPVs, a useful generator must be coupled to filters for absorption, energy alignment, packing, miscibility, and processability, so generative ML should be viewed as candidate-pool expansion followed by screening and active learning.

### 5.4. High-Throughput Screening and Active Learning

High-throughput virtual screening operationalizes ML prediction and generation by ranking large candidate spaces before expensive simulation or synthesis (Figure 3d). The FreMFp + CatBoost workflow screened 6 million donor candidates and identified D18-D2Cl:L8-BO with a predicted PCE of 19.59% [88]. Suthar et al. used random forest, gradient boosting, and SHAP analysis across 1242 donor–acceptor pairs, achieving PCE prediction with r = 0.791 and reporting experimental validation at PCE = 15.23% [99]. Together, these examples show how ML compresses a broad molecular search into a tractable set of targets, although the quantitative values remain model- and dataset-specific.

High-throughput experimentation extends data-driven OPV design from virtual screening to automated fabrication and measurement [100]. The roll-to-roll MicroFactory example shows how thousands of devices can be produced rapidly enough to train predictive models of J-V behaviour and identify dominant processing variables such as donor:acceptor ratio. Its broader significance is not a single device dataset, but the possibility that fabrication workflows themselves can become structured data generators for ML-guided optimization.

Active learning closes the loop by deciding which calculation or experiment should be performed next. The BO-Bagging framework combines Bayesian optimization with bagged decision-tree modelling, with an average reported r = 0.92 and an inference time of 0.00062 s per sample, allowing model predictions to guide experiments in near real time [101]. Transfer learning provides a complementary route when labelled OPV data are limited: a three-stage SchNet workflow reported property-specific MAE reductions in the 32–45% range and narrowed 3710 candidates to 46 candidates [102]. In this role, ML does more than accelerate evaluation; it changes the design sequence from passive database analysis to iterative selection, measurement, and model updating.

For molecular-property prediction, general materials informatics models provide useful methodological anchors, including neural fingerprints, graph convolutional representations, and graph neural networks for chemistry and materials science [103,104,105].

### 5.5. Interpretable ML: Extracting Physical Insight

Interpretability addresses the concern that ML models may predict OPV performance without explaining the structural or processing factors that drive the prediction. SHAP analysis provides one route by assigning Shapley-value contributions that estimate how much each feature contributes to each model output (Figure 3b) [106]. In OPV applications, the cited studies provide source-specific examples: ionization potential and electron affinity are reported as key predictors in one performance model [107]; active device area emerges as a key fabrication variable in a PM6:Y6 processing study [108]; bicyclic or fused-ring fragments correlate positively with PCE in a generative-screening workflow [96]; and substructures such as 4-fluoro-2-methylthiophene and dithienobenzodioxole are associated with donor performance in the FreMFp screening study [88]. These examples illustrate how model interpretation can convert statistical correlations into correlation-informed design hypotheses, while stopping short of treating them as universal design rules [88,96,107,108].

The value of interpretable ML is therefore not limited to explaining a completed prediction. It can close a design loop in which a model predicts performance, SHAP or related tools identify the structural and processing variables responsible, and chemists or experimentalists use those variables to design the next molecular library or processing campaign. This changes the role of ML from merely giving a number to providing a ranked set of hypotheses about why one donor–acceptor composite should outperform another. The resulting cycle links ML, experiment, and chemical intuition, and prepares the ground for multiscale integration, where ML becomes infrastructure for connecting molecular, morphological, and device-level models.

### 5.6. Summary

Machine learning in OPV modelling has evolved from a niche statistical tool into a pervasive design infrastructure. In the progression reviewed here, property-prediction models translate molecular structure into key parameters, generative models invert that relationship to propose new candidates, screening and active learning prioritize the next simulations or experiments, and interpretable ML extracts correlation-informed design hypotheses from learned relationships. The central value of ML in this review is therefore not any single architecture, but its capacity to accelerate specific, well-defined prediction, generation, screening, and interpretation tasks.

Generative and inverse-design methods should be read against the broader molecular-design literature, where continuous latent spaces, high-throughput virtual screening, and generative models have already been used to connect molecular representation with property optimization [109,110,111].

The studies reviewed here do not identify a single ML architecture that is uniformly superior across OPV tasks. Descriptor-based ensemble models remain useful for small experimental datasets; whereas, graph and transformer models benefit from broader and more chemically diverse training data [84,85,87,88,89,90,91,92,93]. Generative models expand the candidate space, but molecular validity does not by itself establish synthesizability, packing compatibility, or device performance [86,96,97,98,109,110,111]. Model claims should therefore be assessed together with data provenance, chemical-domain coverage, uncertainty, and prospective validation.

## 6. Multiscale Integration Frameworks

### 6.1. The Integration Challenge

Multiscale integration in organic photovoltaic modelling is not simply the juxtaposition of molecular, morphological, transport, and device simulations; it is the construction of a reproducible parameter-passing chain. Three problems define this challenge. First, parameter transfer asks how quantities generated at one scale are translated into the variables required at the next scale: molecular structure → electronic parameters → morphology → transport parameters → device parameters → J-V/PCE. This parameter-passing and validation-loop logic is illustrated in Figure 1d.

Second, uncertainty propagation asks where errors accumulate as molecular conformations, site energies, mobilities, and recombination constants are successively coarse-grained. Third, computational tractability asks whether the full workflow can be run within a practical time budget rather than remaining a retrospective explanation.

**Figure 4:**
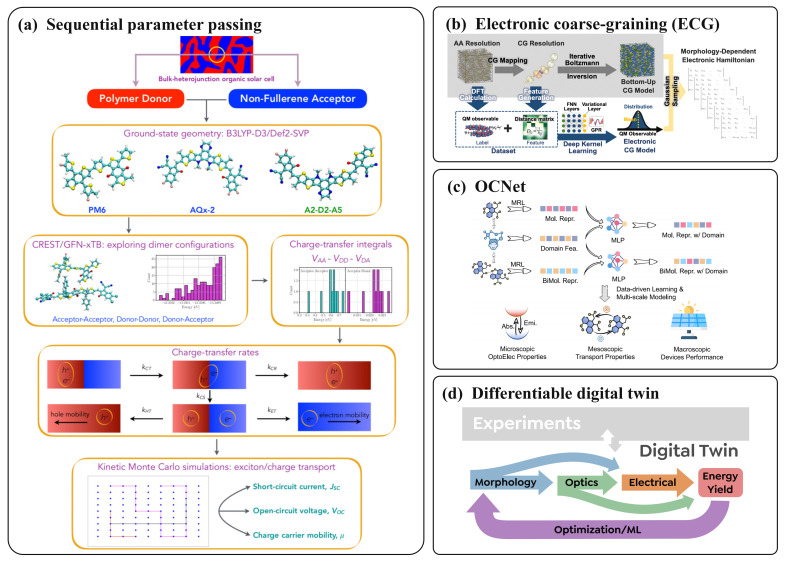
Multiscale integration frameworks for OPV structural design and device modelling. (**a**) Representative sequential parameter-passing workflow in which donor and acceptor molecular structures are optimized, representative dimer configurations are sampled, charge-transfer integrals and rates are estimated, and the resulting parameters are transferred into kinetic Monte Carlo simulations to obtain device-relevant quantities such as short-circuit current, open-circuit voltage, and charge-carrier mobility. (Reproduced with permission from Ref. [112], copyright 2024 American Chemical Society). (**b**) ECG strategy in which atomistic or all-atom configurations are mapped to coarse-grained representations, quantum-mechanical observables are used as training labels, and deep-kernel-learning models generate morphology-dependent coarse-grained electronic Hamiltonians, reducing the need for repeated backmapping and electronic-structure calculations. (Reproduced from Ref. [53], licenced under CC BY 3.0). (**c**) OCNet virtual-characterization framework, where molecular representations and domain descriptors are combined through machine-learning modules to connect microscopic optoelectronic properties, mesoscopic transport properties, and macroscopic device performance. (Reproduced with permission from Ref. [113], distributed under the terms of the Creative Commons Attribution 4.0 International Licence (CC BY 4.0)). (**d**) Differentiable digital-twin concept in which morphology, optical simulation, electrical simulation, and energy-yield prediction are coupled within an optimization/ML loop. Unlike conventional sequential workflows, differentiable frameworks aim to propagate gradients or feedback signals from device-level outputs back toward morphology, material, and processing variables. (Reproduced from Ref. [114]). Together, these approaches illustrate a progression from physically transparent parameter passing toward surrogate-accelerated and optimization-ready multiscale OPV modelling.

Because detailed morphology generation and device simulation are treated in the surrounding sections of the full review, this section focuses on how those modules are connected rather than reintroducing each method. The solutions to these three problems organize the discussion in Section 6.2, Section 6.3, Section 6.4, Section 6.5, Section 6.6 and Section 6.7, from sequential physical pipelines to machine-learning surrogates and differentiable digital-twin concepts. Figure 4 provides a schematic overview of these integration strategies, comparing sequential parameter-passing workflows, ML-accelerated electronic coarse-graining, knowledge-enhanced virtual characterization, and differentiable digital-twin frameworks.

### 6.2. Sequential Parameter-Passing Pipelines

Sequential parameter-passing workflows are useful when they are described according to the scale transition they actually validate [112]. The first-principles-to-kMC example passes molecular and molecular-pair electronic quantities into a statistical kinetic Monte Carlo model to connect charge-transfer parameters with device-relevant quantities such as short-circuit current and open-circuit voltage. It should therefore be framed as a molecular-to-transport bridge, not as a fully validated DFT-to-MD-to-KMC-to-drift-diffusion pipeline with a guaranteed PCE error.

Pipeline variants show that the same logic can be adapted to different material questions without abandoning the sequential structure. Zhou et al. connect DFT, MD, and KMC to examine how alkyl side chains influence aggregation, π-π connectivity, and mobility, with the mobility reported to increase from 2.4 to 3.9 × 10^−4^ cm^2^ V^−1^ s^−1^ [10]. This example keeps the parameter-passing logic local to charge transport: molecular side-chain structure changes packing, packing changes electronic connectivity, and connectivity changes mobility. Sanyam and Mondal extend a comparable multiscale charge-transfer workflow to hole-transporting materials, indicating transferability to neighbouring organic–electronic transport problems rather than proving the same workflow for every OPV active layer [115].

The main limitation of sequential pipelines is that their physical transparency comes with an unfavourable sampling burden. Realistic morphologies contain thousands of molecular pairs, and each pair may require quantum-chemical estimates of site energies, transfer integrals, or reorganization energies before transport can be simulated. As a result, the most detailed workflows can become difficult to deploy for rapid screening, uncertainty quantification, or inverse design. This bottleneck motivates Section 6.3: ML is introduced not as a replacement for the physics-based pipeline, but as a surrogate layer that accelerates expensive parameter evaluations while preserving the same molecular-to-device direction of information flow.

### 6.3. Machine Learning Surrogates as Scale Bridges

ML surrogates function as scale bridges when they replace repeated electronic-structure evaluations while preserving the physical role of each scale [116]. In the SphereNet strategy, MD supplies thermally accessible dimer configurations, DFT supplies transfer-integral targets, and the GNN accelerates the mapping from morphology-derived geometry to electronic coupling. The important caution is that the surrogate does not remove the need for physical validation; training coverage and the specific mobility response reported in the source should not be generalized into a broad claim of large mobility change.

Transfer learning provides a complementary route when direct DFT-labelled data are limited. A model pretrained on QT packing data can be fine-tuned with a smaller number of DFT calculations for the target morphology-mobility problem, reducing the amount of new quantum chemistry required [117]. The two strategies can be read as an interpretive cost-data trade-off: SphereNet-style models emphasize high-fidelity transfer-integral prediction from target configurations; whereas, transfer learning reduces new labelled-data requirements by reusing information from an upstream morphology-related task [116,117]. In both cases, ML functions as a scale bridge, converting molecular geometry and morphology-derived configurations into transport-relevant electronic parameters more efficiently than brute-force electronic-structure sampling.

### 6.4. Knowledge-Enhanced Virtual Characterization: OCNet

Knowledge-enhanced virtual characterization differs from generic property prediction by combining learned representations with descriptors already tied to OPV physics [113]. OCNet exemplifies this direction by fusing equivariant molecular features with TB-level electronic descriptors, overlap integrals, transfer-integral information, and structural angle/distance descriptors. Its reported improvement over TD-DFT-descriptor-only prediction and large cost reduction support the value of physics-informed feature fusion, while the following paragraph clarifies why this remains distinct from a fully differentiable physical pipeline.

The methodological significance of OCNet is the “knowledge-enhanced” modelling philosophy. Rather than asking a deep-learning model to rediscover electronic, coupling, and structural descriptors only from sparse performance labels, the architecture explicitly encodes these physical priors and reserves the neural network for the nonlinear mapping from molecular representation to device response. This places OCNet between conventional sequential simulation and fully differentiable simulation. OCNet is an end-to-end molecular-to-PCE predictor; whereas, Sol(Di)^2^T, discussed in Section 6.5, is a differentiable physical-pipeline concept; the two directions are therefore complementary rather than redundant, although the distinction between the two directions should be kept explicit [113,114].

### 6.5. Toward Fully Differentiable Digital Twins

Differentiable digital-twin proposals represent a possible next stage beyond sequential simulation, because gradients can in principle connect target performance back to controllable inputs [114]. The Sol(Di)^2^T framework links morphology, optical simulation, drift-diffusion, and energy-yield modules while replacing selected expensive calculations with neural surrogates. Its significance lies in the differentiable architecture: if implemented and validated, composition, processing, and thickness variables could be optimized against device-level objectives. It should therefore be framed as a prospective design engine rather than a demonstrated manufacturing solution.

Its manufacturing relevance is a future extrapolation rather than a demonstrated industrial result. A practical roll-to-roll (R2R)-oriented digital twin could eventually map how composition, morphology, film thickness, and processing variation influence yield, performance distributions, and robustness, but this remains a future direction until validated in manufacturing-relevant workflows [114]. In the parameter-passing language of this section, the digital-twin concept closes the loop: gradients travel backward from J-V/PCE or energy yield to device, transport, morphology, electronic, and molecular or processing variables.

### 6.6. Cross-Scale Case Study: Fluorination

Fluorination is useful here as a cross-scale case study because the same molecular modification influences descriptors introduced in Section 2, Section 3 and Section 4, including electronic coupling, molecular packing, morphology, transport, and device output [49]. The staged chain runs from backbone compression and reduced vibrational relaxation, through planarity, reorganization energy, and transfer integrals, to crystallization, domain size, percolation, and device output. The numerical PCE comparisons should remain clearly separated across the PM6:Y5-to-PM6:Y6 and PM6:Y16-to-PM6:Y18 examples; PM6 is the polymer donor; whereas, Y5, Y6, Y16, and Y18 are Y-series non-fullerene acceptors; the next paragraph then draws the broader multiscale-design lesson from this case.

This case study illustrates why single-scale analysis is incomplete for OPV structural design. Fluorination can be interpreted through a connected cascade from sub-angstrom backbone changes to molecular packing, crystallization, percolation, and device response, but the claim that this cascade provides a complete mechanistic picture should be treated as an interpretive synthesis rather than a direct source finding [49]. The purpose of multiscale modelling is therefore not merely to fit a final device metric, but to identify which link in the chain converts chemical substitution into improved charge generation, transport, and collection. In this sense, fluorination is a template for how future OPV design claims should be tested: each proposed molecular modification should be traced through electronic parameters, morphology, transport, and device response rather than evaluated only by final PCE.

### 6.7. Cross-Scale Comparison and Method-Selection Guidelines

The comparisons in Table 1, Table 2, Table 4, Table 5, Table 6 and Table 7 show that no method spans all relevant OPV scales without a trade-off between resolution, sampling, and parameterization. Quantum and atomistic methods provide mechanistic detail but are limited in system size and timescale [22,23,24,25,26,39,40,51,52]. Coarse-grained, phase-field, KMC, and drift-diffusion models access larger scales, but introduce increasingly explicit assumptions about mapping, rate laws, disorder, and constitutive parameters [20,47,58,59,60,61,62,63,64,68,69,70,71,72,73,74,75,76,77,78,79,80]. ML surrogates can reduce repeated calculations, although their reliability remains bounded by training-domain coverage and label quality [84,85,86,87,88,89,90,91,92,93,94,95,96,97,98,99,100,101,102,103,104,105,106,113,116,117].

Based on these comparisons, method selection should begin with the quantity required by the next scale and the independent observable available for validation. Table 8 consolidates the principal role, strengths, limitations, and validation routes of the major approaches. It is intended as a decision aid rather than a universal ranking: the appropriate model depends on the scientific question, data availability, and acceptable uncertainty.

We therefore recommend a modular rather than maximally complex workflow. Each added module should supply an identifiable downstream parameter, and its assumptions should be testable with at least one independent observable. Preserving parameter provenance and uncertainty across interfaces is more important than adding model complexity that cannot be validated.

## 7. Experimental Validation

### 7.1. Morphology Characterization and Like-for-Like Validation

Morphology validation should compare each simulated structural level with the experimental observable that actually probes it [2]. GIWAXS/grazing-incidence X-ray diffraction (GIXD) constrains packing and orientation, RSoXS probes compositional length scales, and AFM, TEM, or photoinduced force microscopy (PiFM) provide surface or real-space chemical contrast. The double-fibril morphology example is useful because its domain spacing and carrier length scales can be compared quantitatively, but the transferable lesson is not a universal optimum size; it is the need to match simulated morphology descriptors to measurable length scales.

These techniques are complementary rather than interchangeable. DeLongchamp and co-workers noted that grazing-incidence scattering is sensitive to orientation and buried structure but depends on geometry and model-based interpretation; TEM contrast is affected by staining, thickness, and beam damage; AFM mainly probes the surface; XPS and neutron reflectometry are better suited to vertical composition [118]. A simulated structure factor should be compared with scattering, while a simulated height or phase map can be compared with AFM. Placing a three-dimensional composition field beside a micrograph and judging visual similarity is not quantitative validation.

### 7.2. Spectroscopic Validation of Charge-Generation Dynamics

Spectroscopic validation constrains the time scales and populations that transport models often treat as fitted rates [18]. Across blends with different HOMO offsets, the cited measurements show that negligible energetic offset can slow exciton splitting and reduce EQE unless the exciton lifetime is long enough to compensate. Such data can test dissociation times in KMC or rate equations directly, instead of validating a model only through the final free-charge yield.

NAMD-derived intermediate states become most useful when they are translated into spectroscopic predictions [22]. The X-SH mechanism discussed earlier predicts a short-lived, partly delocalized XT-CT hybrid state before non-interfacial CT and charge-separated states form. The testable claim is therefore not simply that delocalization helps, but that a hybrid state with a specific time window, spatial extent, and energy should be sought in ultrafast spectra.

### 7.3. Device-Level Validation and Model-Failure Localisation

J-V and EQE are necessary integrated benchmarks, but they do not identify a model uniquely. Mobility, recombination coefficient, series resistance, and contact barriers can compensate for one another, so similar curves may represent different internal physics. A more defensible sequence constrains each module independently. Optical constants, absorption, and thickness first constrain generation. SCLC, time-resolved microwave conductivity (TRMC), time-of-flight (TOF), or transient current then constrain mobility. Light-intensity and temperature trends, TPV, charge extraction, and impedance constrain recombination and contacts. Only after these checks should the complete J-V, EQE, and open-circuit-voltage loss be compared.

An auditable workflow should also make failure locatable. If a TMM-derived G(x) disagrees with absorption or EQE, n, k, and layer thickness are checked first. If a morphology structure factor disagrees with scattering, the force-field or phase-field parameters are revisited. If mobility has the wrong temperature or density dependence, the DOS and coupling model require attention rather than a compensating change in series resistance. Agreement in PCE becomes explanatory only after the constituent modules are defensible against independent data.

### 7.4. Validation Gaps, Uncertainty, and Reporting Requirements

Agreement with experiment does not establish that the parameters are uniquely identified. Local sensitivity addresses small perturbations around a selected solution; whereas, global sensitivity is more appropriate for broad parameter ranges and nonlinear interactions. In either case, parameter correlations, confidence intervals, or acceptable parameter ensembles should accompany a best-fit value. When several parameter combinations produce nearly identical J-V curves, the defensible conclusion concerns an identifiable combination or trend, not a unique microscopic parameter.

Calibration data should also be separated from validation data. A practical hold-out test may fit a subset of thicknesses and predict another thickness, calibrate at room temperature and predict the temperature dependence, or determine recombination from one light-intensity range and test another. Table 7 lists minimum reporting information for each module. The checklist cannot remove model discrepancy, but it allows a reader to determine which measurements, assumptions, and numerical settings support the conclusion.

Most validation is performed after film formation and consequently observes only the final state left by the processing history. Establishing the sequence of solvent evaporation, nucleation, demixing, and crystallization requires in situ or rapidly time-resolved absorption, GIWAXS, RSoXS, and optical microscopy, with the experimental time resolution matched to the model time scale. Similar final morphologies need not have followed the same path, and different paths may leave different interfacial purity, residual stress, and long-term stability.

The operating morphology is also not a static input. Continuous illumination, temperature cycling, oxygen and water ingress, and bending can drive coarsening, component migration, crystal rearrangement, delamination, and trap growth. Materials models should move from predicting initial efficiency to predicting how descriptors drift with time, tested against aged GIWAXS/RSoXS, EQE, J-V, impedance, and mechanical cycling. Translation from spin coating to blade coating, slot-die coating, or larger-area devices should likewise include flow, drying gradients, and batch variation; otherwise, an apparently optimal morphology may be specific to small-area, slowly and uniformly dried films.

Most current multiscale studies remain largely retrospective. Parameters are fitted to a target device and then used to explain that same device. A prospective calculation would generate a morphology distribution from molecular structure and processing, propagate uncertainty through transport, and predict performance without direct fitting to the target J-V curve. Cross-batch data, common data interfaces, and routine reporting of negative results and parameter correlations are still missing from that workflow.

A practical near-term objective is therefore a closed validation loop within one material system: explicit provenance and uncertainty for each input, at least two independent experimental constraints, sensitivity or identifiability analysis for parameters that control the conclusion, and a clear distinction between data-supported findings and model-dependent inference. Such a study may not span every scale at once, but it can establish the model’s domain of validity, locate failure, and define reusable interfaces for subsequent cross-material prediction.

## 8. Challenges, Opportunities, and Outlook

### 8.1. Persistent Challenges

Accuracy vs. computational cost. The fundamental tension in multiscale OPV modelling is between the accuracy of high-level quantum chemical methods (coupled-cluster, multireference methods) and the computational cost that limits them to small model systems, versus the efficiency of semi-empirical or classical methods that may miss essential physics. Recent NAMD and X-SH studies illustrate the frontier of this trade-off, but they still operate on atomistic models and short excited-state trajectories that remain far below the length and time scales needed to follow full morphology evolution [22,23].

Disorder. Energetic and structural disorder is not a perturbation in organic semiconductors—it is a defining characteristic. Accurately modelling the Gaussian density of states, the spatial correlations of energetic disorder, and their consequences for charge transport remains challenging at every scale. The PRX study by Stojanović et al. [9] demonstrating that electrostatic disorder in acceptor-donor–acceptor (A-D-A)-type NFAs reduces mobility by 4–5 orders of magnitude highlights both the importance and the difficulty of the disorder problem.

Transferability. Force fields, exchange-correlation functionals, and ML models developed for one chemical system often fail when applied to another. The extraordinary diversity of OPV donor and acceptor chemistries—from semicrystalline polymers to amorphous small molecules—resists the development of universal parameterizations.

Stability and degradation. Compared with initial-performance modelling, degradation-aware multiscale modelling—photo-oxidation, morphological instability, interfacial reactions, and mechanically induced failure—remains less developed and represents a critical gap because operational lifetime, not only initial efficiency, is a barrier to commercialization [15].

### 8.2. Opportunities for Cross-Disciplinary Fertilization

Several specific opportunities exist where composites-domain expertise could accelerate OPV development:

Topology optimization. The composite structural design community has developed sophisticated topology optimization methods for designing load-bearing structures with optimal material distribution. Analogous methods could be applied to optimize the spatial distribution of donor and acceptor phases in the BHJ—a “topology optimization of the nanoscale composite”—to simultaneously maximize exciton harvesting and charge collection.

Multiscale homogenization. The mathematical framework of computational homogenization, which bridges microscale constituent behaviour to macroscale effective properties in structural composites, is directly applicable to the OPV multiscale problem. Homogenization theory could provide rigorous bounds on effective mobility and recombination coefficients as functions of morphology, replacing the current practice of ad hoc averaging.

Damage mechanics and lifetime prediction. The composites community has decades of experience in modelling progressive damage accumulation (matrix cracking, fibre breakage, delamination) under cyclic and environmental loading. Adapting these frameworks to OPV degradation—where “damage” manifests as trap formation, phase demixing, and interfacial delamination—could provide a systematic approach to lifetime prediction that is currently lacking.

Uncertainty quantification (UQ). The stochastic nature of BHJ morphology and the sensitivity of device performance to processing conditions make UQ essential for any model intended to guide manufacturing. Bayesian UQ frameworks, widely used in aerospace composites, could quantify the confidence intervals on predicted PCE and identify the dominant sources of variance.

### 8.3. Emerging Directions

Physics-informed machine learning. The next generation of ML models for OPV will likely embed physical constraints—symmetries, conservation laws, thermodynamic consistency—directly into model architectures, reducing data requirements and improving extrapolation to unseen chemistries. Equivariant neural networks provide an OPV-relevant example of this trend [113]; more explicit Hamiltonian or conservation-law constraints remain a prospective extension rather than an established OPV workflow.

Pretrained molecular models for organic electronics. Transformer-style molecular pretraining and knowledge-enhanced virtual characterization suggest that OPV-specific fine-tuning can complement task-specific fingerprints and descriptor sets, especially when paired with domain descriptors and experimental feedback [85,113].

ML-guided data production and optimization. Current OPV evidence points more directly to roll-to-roll-compatible data production and ML models for predicting device behaviour than to fully autonomous OPV self-driving laboratories [100]. Such data infrastructure could become a prerequisite for future closed-loop optimization, but that step should be treated as prospective.

Digital twins for manufacturing. Extending the Sol(Di)^2^T concept [114] from laboratory-scale devices toward R2R manufacturing environments could eventually support manufacturing-facing digital twins that estimate yield, performance distributions, and process-window sensitivity. At present, this remains a forward-looking extrapolation from differentiable solar-cell modelling rather than a validated R2R manufacturing workflow.

### 8.4. Concluding Remarks

The bulk heterojunction active layer of an organic solar cell can be productively viewed as a nanostructured composite material. Its performance depends on molecular-level engineering of donor/acceptor interfaces, mesoscale control of phase-separated morphology, and device-scale optimization of optical and electrical architectures. These coupled design variables demand a broad set of multiscale numerical modelling tools. Over the past five years, the OPV community has built a diverse simulation ecosystem spanning quantum dynamics, molecular dynamics, kinetic Monte Carlo, drift-diffusion, and machine learning. The non-fullerene acceptor revolution has raised reported single-junction efficiencies toward, and in some reports above, the 20% threshold [1,2,3] while exposing the limitations of classical modelling paradigms, driving the development of new methods—NAMD, dKMC, electronic coarse-graining, generative ML—that explicitly treat quantum delocalization, system-specific packing, and multiscale coupling in modern OPV materials.

This review surveys this field systematically, from molecular orbitals to device J-V curves, with particular attention to the frameworks that connect the scales. It also highlights the structural-design perspective that positions OPV active layers within the broader conceptual framework of composite materials, and this framing can encourage productive engagement between the OPV and structural composites research communities.

The challenges that remain—predictive simulation of novel chemistries, multiscale modelling of degradation, uncertainty-aware design optimization—are substantial but tractable. As physics-informed machine learning, ML-guided experimentation, and differentiable simulation converge, a more integrated computational pipeline for rational OPV nanocomposite design—from molecular sketch toward manufactured device—becomes a concrete research target.

## Figures and Tables

**Table 1:** Non-adiabatic molecular-dynamics approaches discussed for OPV interfacial charge generation and exciton transport.

Method	Representative Work	Scale and Time Window	Main Physics and Boundary
TSH	Tully et al. 1990 [39]	Small molecular systems; short non-adiabatic trajectories	Stochastic transitions among electronic states; sensitive to state definitions, decoherence treatment, and sampling
Ehrenfest MD	Liu et al. 2024 [23]	Ultrafast interfacial dynamics over tens of femtoseconds	Mean-field electron-nuclear coupling; electron-nuclear dynamics remain mean-field
X-SH	Ivanović et al. 2025 [22]	Nanoscale interfacial models; trajectories up to approximately 10 ps	Coupled Frenkel-exciton and CT-state configurations; high computational cost
NAMD	Stojanovic et al. 2024 [40]	Molecular crystal/thin-film models; short excited-state trajectories	Exciton transport and disorder effects; system-specific, not a universal NFA-blend morphology model

**Table 2:** Mesoscale morphology and morphology-to-transport modelling methods, direct outputs, and scale-transfer risks.

Method	Resolution and Scale	Direct Outputs	Main Risk and Best Use
AA-MD	Atoms; typically ns to 100 ns	Packing, conformations, contacts	Limited box size and sampling; best for interfacial chemistry and local structure
CG-MD	Beads; larger boxes and microsecond-scale trajectories	Domains, connectivity, aggregation statistics	Accelerated dynamics require validation or backmapping; best for statistical morphology and processing trends
Electronic coarse-graining (ECG)	CG morphology; tens-of-nanometers morphology	Site energies, electronic couplings, coarse-grained Hamiltonians	Training coverage and extrapolation control reliability; best for linking morphology to electronic parameters
Phase field	Continuum; approximately 100 nm and long times	Composition, crystallinity, structure factor	Parameters are hard to measure and molecular contacts are absent; best for drying, annealing, and domain evolution
KMC	Site network; mesoscale transport	Mobility, diffusion length, generation/recombination rates	Depends on energy mapping and rate law; best for morphology-to-device parameter transfer

**Table 3:** Controllable materials variables, structural descriptors, associated properties, and validation methods.

Controllable Variable	Primary Structural Descriptor	Associated Property	Suggested Validation
Solvent, solid additive, and evaporation rate	Mixing, nucleation sequence, interfacial width, domain size, vertical segregation	Exciton access, phase purity, connectivity	In situ absorption/scattering, RSoXS, cross-sectional composition
Thermal or solvent annealing	Crystallinity, crystal stability, fibril width, coarsening rate	Electronic coupling, mobility, recombination, morphological stability	In situ GIWAXS, AFM/TEM time series, temperature-dependent transport
Backbone, end-group, and side-chain structure	Conformational rigidity, packing orientation, electrostatic disorder, compatibility	DOS width, transfer integrals, reorganization energy, dielectric response, trap distribution	GIWAXS, UPS/IPES, dielectric spectroscopy, temperature-dependent mobility
Active-layer thickness and interlayers	Optical field, vertical field, vertical composition, contact selectivity, space charge	Short-circuit current, open-circuit voltage, FF, extraction loss	Ellipsometry/absorption, thickness series, dark/illuminated J-V, impedance
Light, heat, oxygen, water, and mechanical stress	Coarsening, component migration, crystal rearrangement, interface degradation	Mobility drift, trap growth, lifetime loss	Operando GIWAXS/RSoXS, aged EQE/J-V, spectroscopy, mechanical cycling

**Table 4:** Drift-diffusion inputs, independent constraints, validation observables, and common identifiability problems.

DD Input/Module	Independent Constraint	Validation Observable	Common Identifiability Problem
Optical generation profile G(x, λ)	n, k, thickness, TMM/FDTD	Absorption, EQE, thickness-dependent photocurrent	Optical errors compensated by collection efficiency
Mobility μ(n, F, T)	SCLC, transients, KMC/EGDM	Temperature-, field-, and density-dependent J-V or transient current	Constant mobility hides DOS errors
Recombination R(n, *p*)	TPV, CE, transient current	Lifetime-density and light-intensity trends	Tail states compensate contact losses
DOS and tail states	UPS/IPES, temperature transport, constrained fit	Ideality factor, carrier density, temperature trends	Gaussian and exponential tails may fit similar steady-state curves
Contacts/interfaces	Work functions and selective-layer data	Dark J-V, impedance, thickness dependence	Bulk parameters absorb contact errors

**Table 5:** Scope and boundaries of commonly used optical and device-simulation platforms.

Platform	Core Model	Suitable Question	Main Boundary and Reference
TMM-Sim	TMM optics	Planar optical field, G(x), parasitic absorption	No lateral texture or full carrier transport [13]
OghmaNano (formerly gpvdm)	TMM/ray tracing plus DD plus DOS/transients	OPV optoelectronic coupling and parameter scans	Strong dependence on material parameters [14,71,72]
SETFOS	Optics plus DD	Commercial calibration and steady/transient fitting	Commercial licence; model choice still requires judgement [72]
SIMsalabim	One-dimensional DD	Rapid reproducible device models and batch scans	One-dimensional homogeneous approximation [79]
SolarDesign	Materials-device-circuit multiphysics	Online cross-level design and teaching	Database and specialized-model assumptions require checking [80]

**Table 6:** Representative machine-learning property-prediction approaches for OPV donor–acceptor materials.

Method	Representative Model	Target and Reported Metric	Main Advantage and Boundary
Fixed molecular descriptors	Dragon + GBRT	PCE; R^2^ = 0.84 for small molecules and 0.69 for polymers [87]	Works with sparse experimental data and does not require HOMO/LUMO as direct inputs; fixed descriptors may miss NFA-specific structural motifs
Task-adapted molecular fingerprint	PUFp + RF	PCE for NFA materials; outperformed MACCS fingerprints [84]	Encodes OPV-relevant polymer-unit fragments; transferability depends on whether new candidates share the learned fragment chemistry
Frequency molecular fingerprint	FreMFp + CatBoost	PCE; R^2^ = 0.82 [88]	Enables large-scale candidate ranking; predicted high-PCE candidates require higher-level computation or experimental validation
SLI-GNN + LightGBM	SLI-GNN + LightGBM	PCE; Pearson r = 0.79, validated errors below 2% [89]	Learns molecular-graph information and has experimental follow-up; performance remains dataset- and device-context-dependent
Ring-aware graph representation	RingFormer	OSC molecular-property; 22.77% relative improvement on the source benchmark [90]	Captures atom-, ring-, and inter-ring-level structure; benchmark improvement should not be treated as universal OPV accuracy
Transformer	abcBERT	PCE/electronic properties; HOMO MAE = 0.057 eV [85]	Uses large calculated datasets for pretraining and smaller experimental datasets for finetuning; errors are benchmark-specific rather than general DFT
TD-DFT-trained graph model	RGNN	HOMO/LUMO; R^2^ approximately 0.84–0.85 [92]	Useful for fast electronic-property screening; calculated-label accuracy does not directly imply device-performance accuracy
Ensemble learning	SMILES + BoostedTree	PCE; R^2^ = 0.8526 [93]	Provides a practical small-data baseline by combining molecular-string descriptors with ensemble regression; reliability remains dataset-dependent

**Table 7:** Minimum reporting information and suggested uncertainty checks for validating multiscale OPV models.

Module	Minimum Information to Report	Suggested Uncertainty Check
Optics	n, k, layer thicknesses, spectral range, boundary conditions	Vary thickness and optical constants; inspect absorption/EQE residuals
Morphology	Box size, replicas, descriptor definitions, mapping or phase-field parameters	Report distributions across independent morphologies and finite-size effects
Transport	DOS, rate law, field/temperature/density dependence of mobility	Compare rate laws and perform local or global sensitivity analysis
Device	Contacts, recombination, mesh, solver tolerances, initial conditions	Fit several data sets jointly; inspect parameter correlation/non-uniqueness
Cross-module validation	Separation of calibration data from independent test data	Hold out one thickness, temperature, or light-intensity condition

**Table 8:** Cross-scale comparison and selection guidelines for major OPV modelling approaches.

Method	Primary Role and Strength	Main Limitation or Risk	Recommended Use, Validation, and Representative References
DFT/TD-DFT	Electronic levels, couplings, and excited-state classification with chemical interpretability.	Functional and geometry sensitivity; limited interfacial sampling.	Use for molecular/interface descriptors; validate against spectroscopy and electrochemical data [19,24,25,26,27,28,29,30,31,32,33,34,35,36,37,38].
NAMD/X-SH	Real-time exciton and charge-transfer dynamics with explicit electron-nuclear coupling.	High computational cost, short trajectories, and limited ensemble sampling.	Use for ultrafast pathways; validate with time-resolved spectroscopy [22,23,39,40].
AA-MD	Atomistic packing, conformations, and local interfacial contacts.	Limited box size and timescale for full film formation.	Use for local structure and interface chemistry; validate with packing-sensitive measurements [4,51,52].
CG-MD	Larger morphology ensembles, aggregation, and connectivity statistics.	Mapping and force-field transferability; electronic detail is lost without backmapping or surrogates.	Use for morphology trends; validate with scattering and atomistic backmapping [4,12,51,52,53].
Phase field	Drying, annealing, crystallization, and domain evolution over larger scales.	Phenomenological thermodynamic and kinetic parameters; no molecular contacts.	Use for processing history; validate with time-resolved morphology data [11,59,63,64].
KMC/dKMC/jKMC	Morphology-to-transport mapping, including localized or partially delocalized motion.	Sensitive to site definitions, rate laws, disorder mapping, and localization assumptions.	Use for mobility, diffusion, and recombination; validate against transport and lifetime measurements [9,19,20,47,58,60,61,62].
TMM/FDTD	Optical field, absorption, and generation profiles.	TMM assumes planar layers; FDTD requires detailed geometry and may omit electrical losses.	Use for optical source terms; validate against absorption and EQE [13,65,66,67].
Drift-diffusion	Device-scale J-V, EQE, extraction, and loss analysis.	Parameter non-uniqueness and compensation among transport, recombination, and contacts.	Use when inputs are independently constrained by optical, steady-state, and transient data [14,68,69,70,71,72,73,74,75,76,77,78,79,80].
ML and surrogates	Rapid prediction, screening, inverse design, and acceleration of expensive mappings.	Dataset bias, extrapolation risk, uncertain labels, and limited prospective validation.	Use within a defined chemical domain; report uncertainty and validate prospectively [83,84,85,86,87,88,89,90,91,92,93,94,95,96,97,98,99,100,101,102,103,104,105,106,113,116,117].

## Data Availability

No new data were created or analyzed in this study. Data sharing is not applicable to this article.

## Abbreviations

AA-MD	all-atom molecular dynamics
A-D-A	acceptor-donor–acceptor
A-DA’D-A	acceptor-donor–acceptor’-donor–acceptor
AFM	atomic force microscopy
AIMD	ab initio molecular dynamics
BHJ	bulk heterojunction
BTBT	benzothieno [3,2-b]benzothiophene
CE	charge extraction
CG-MD	coarse-grained molecular dynamics
CS	charge separated
CT	charge transfer
D18	donor material
D18-D2Cl	donor material
D/A	donor/acceptor
DCBB	3,5-dichlorobromobenzene
DD	drift-diffusion
DFT	density functional theory
DIO	1,8-diiodooctane
dKMC	delocalized kinetic Monte Carlo
DOS	density of states
DRCN5T	donor material
ECDM	extended correlated disorder model
ECG	electronic coarse-graining
EGDM	extended Gaussian disorder model
EQE	external quantum efficiency
ETL	electron-transport layer
F-BTA3	guest acceptor and third component
FDTD	finite-difference time-domain
FF	fill factor
FreMFp	frequency molecular fingerprint
GBRT	gradient-boosted regression trees
GIWAXS	grazing-incidence wide-angle X-ray scattering
GIXD	grazing-incidence X-ray diffraction
GNN	graph neural network
HOMO	highest occupied molecular orbital
HTL	hole-transport layer
IPES	inverse photoelectron spectroscopy
IQE	internal quantum efficiency
ITO	indium tin oxide
jKMC	jumping kinetic Monte Carlo
JSC	short-circuit current density
J-V	current density-voltage
KMC	kinetic Monte Carlo
L8-BO	acceptor material
LE	local exciton
LightGBM	Light Gradient Boosting Machine
LSTM	long short-term memory
LUMO	lowest unoccupied molecular orbital
MAE	mean absolute error
MD	molecular dynamics
ML	machine learning
MLJ	Marcus–Levich–Jortner
N3-BO	acceptor material
NAMD	non-adiabatic molecular dynamics
NFA	non-fullerene acceptor
niCT	non-interfacial charge transfer
O-IDTBR	acceptor material
OPV	organic photovoltaic
PC61BM	fullerene acceptor
PC71BM	fullerene acceptor
PCE	power conversion efficiency
PDI	perylene diimide
PiFM	photoinduced force microscopy
PM6	donor material
PUFp	polymer unit fingerprint
PURS	Python-based polymer-unit-recognition script
PYIT	polymer acceptor
R2R	roll-to-roll
RF	random forest
RGNN	residual-gated graph neural network
RSoXS	resonant soft X-ray scattering
SCLC	space-charge-limited current
SHAP	Shapley additive explanations
SLI-GNN	self-learning-input graph neural network
SMILES	simplified molecular-input line-entry system
TD-DFT	time-dependent density functional theory
TEM	transmission electron microscopy
TMM	transfer-matrix method
TOF	time-of-flight
TPV	transient photovoltage
TRMC	time-resolved microwave conductivity
TSH	trajectory surface hopping
UPS	ultraviolet photoelectron spectroscopy
UQ	uncertainty quantification
VOC	open-circuit voltage
X-SH	exciton-state-based surface hopping
XT	excitonic configuration
Y16	acceptor material
Y18	acceptor material
Y2CF3	acceptor material
Y5	acceptor material
Y6	acceptor material
Blend notation: Colons separate the components of binary and ternary blends (e.g., PM6:Y6 and D18:N3-BO:F-BTA3).	
